# Styptic Cotton in Otorrhœa

**Published:** 1876-03

**Authors:** 


STYPTIC COTTON IN THE TREATMENT OF OTORRHŒA.—Dr. Thomas G. Morton, of Philadelphia, writes to the *Medical Times* that he has used styptic cotton with excellent results in a number of cases of discharge from the ears.

The styptic cotton is prepared as follows: Take a roll of fine jeweler's cotton and thoroughly saturate in a mixture of Monsel's solution of the subsulphate of iron, diluted with two parts of water; let it stand in the mixture for forty-eight hours; press the liquid out, and dry in a warm room, then pick or card out in fine shreds. It is better to make it in small quantities, as there seems to be some change in the cotton when kept for any length of time, losing its texture and breaking up in a fine powder when handled, thus rendering it unfit for application. In the case cited, the first plug was removed the day following its insertion, showing a healthier condition of the meatus, with marked lessening of the discharge; all the pus was absorbed by the cotton, which left the drum and canal quite clean; at the end of the first week only a slight moisture remained, and in a fortnight the cure was complete, and has so continued; the opening in the tympanum closed up at the same time.

Encouraged by success in this instance, he has since used no other treatment in a very large number of cases of otorrhœa, and always with the same excellent result.

Before using the cotton, the ear should each time be thoroughly cleansed with tepid water, with a Davidson's syringe, and the canal then wiped out with common cotton; then a portion of the "styptic cotton" should be rolled upon an instrument having a screw-shaped extremity, an illustration of which accompanies the original article. The plug of cotton being at least the length and width of the canal and carried in until the plug rests close to, but not in contact with, the drum; by reversing the screw movement the instrument is easily withdrawn, leaving the cotton behind.

The first few plugs should be changed daily, then, according to the lessening of the discharge, not so frequently.

He has not observed any trouble or pain arise from the use of the styptic cotton, while it usually has an anodyne effect.

(In preparing this cotton, a precaution not mentioned by Dr. Morton consists in carefully cleansing it with caustic soda to remove adherent oil, and subsequent washing to get rid of the alkali.)—*New Remedies, Jan.* 1876.

